# Proctitis 1 Week after Stereotactic Body Radiation Therapy for Prostate Cancer: Implications for Clinical Trial Design

**DOI:** 10.3389/fonc.2016.00167

**Published:** 2016-07-20

**Authors:** Ima Paydar, Robyn A. Cyr, Thomas M. Yung, Siyuan Lei, Brian Timothy Collins, Leonard N. Chen, Simeng Suy, Anatoly Dritschilo, John H. Lynch, Sean P. Collins

**Affiliations:** ^1^Department of Radiation Medicine, Georgetown University Hospital, Washington, DC, USA; ^2^Department of Urology, Georgetown University Hospital, Washington, DC, USA

**Keywords:** prostate cancer, SBRT, CyberKnife, time point, recall period, symptom management trial, EPIC

## Abstract

**Background:**

Proctitis following prostate cancer radiation therapy is a primary determinant of quality of life (QOL). While previous studies have assessed acute rectal morbidity at 1 month after stereotactic body radiotherapy (SBRT), little data exist on the prevalence and severity of rectal morbidity within the first week following treatment. This study reports the acute bowel morbidity 1 week following prostate SBRT.

**Materials and methods:**

Between May 2013 and August 2014, 103 patients with clinically localized prostate cancer were treated with 35–36.25 Gy in five fractions using robotic SBRT delivered on a prospective clinical trial. Bowel toxicity was graded using the Common Terminology Criteria for Adverse Events version 4.0 (CTCAEv.4). Bowel QOL was assessed using the EPIC-26 questionnaire bowel domain at baseline, 1 week, 1 month, and 3 months. Time-dependent changes in bowel symptoms were statistically compared using the Wilcoxon signed-rank test. Clinically significant change was assessed by the minimally important difference (MID) in EPIC score. This was defined as a change of 1/2 standard deviation (SD) from the baseline score.

**Results:**

One-hundred and three patients with a minimum of 3 months of follow-up were analyzed. The cumulative incidence of acute grade 2 gastrointestinal (GI) toxicity was 23%. There were no acute ≥ grade 3 bowel toxicities. EPIC bowel summary scores maximally declined at 1 week after SBRT (−13.9, *p* < 0.0001) before returning to baseline at 3 months after SBRT (+0.03, *p* = 0.94). Prior to treatment, 4.9% of men reported that their bowel bother was a moderate to big problem. This increased to 28.4% (*p* < 0.0001) 1 week after SBRT and returned to baseline at 3 months after SBRT (0.0%, *p* = 0.66). Only the bowel summary and bowel bother score declines at 1 week met the MID threshold for clinically significant change.

**Conclusion:**

The rate and severity of acute proctitis following prostate SBRT peaked at 1 week after treatment and returned to baseline by 3 months. Toxicity assessment at 1 week can therefore minimize recall bias and should aid in the design of future clinical trials focused on accurately capturing and minimizing acute morbidity following SBRT.

## Introduction

Proctitis after prostate cancer radiation therapy is a primary determinant of quality of life (QOL) and remains an ongoing clinical challenge. Acute radiation proctitis commonly peaks during conventionally fractionated treatment (1–4) and resolves a few weeks to months after the completion of therapy (5, 6). Patients with acute radiation proctitis describe symptoms of bowel frequency and urgency, rectal pain, or rectal bleeding (7). Four to 50% of patients treated with conventionally fractionated intensity-modulated radiation therapy (IMRT) develop grade 2 or greater acute proctitis (8–11). The risk of acute radiation proctitis is dependent upon the rectal volume exposed to a high radiation dose (12, 13) as well as the fractionation schema (14). In an attempt to reduce radiation related bowel morbidity, pretreatment dietary intervention (15) or enemas (16) have been utilized with only modest improvements. Several studies have demonstrated that acute rectal symptoms may also be the best predictor of late proctitis (17–19).

Patient-reported outcomes have become an increasingly reliable tool when comparing the relative morbidity of the different treatment options for localized prostate cancer (5). In such a situation, it is important to select the most appropriate time point and recall period for symptom assessment as these time points will vary depending on the evaluated symptom and its acute or chronic nature. Not surprisingly, the rate of memory decay is impacted by the severity and temporal pattern of the symptom, with recall of mild and transient symptoms decaying faster than those of severe and persistent symptoms (20). Thus, assessment of toxicities too far after their occurrence may result in underestimation of their overall incidence in a given treatment group, and the differences between treatment groups may therefore be artificially minimized (20). An early time point with a short recall period, therefore, is generally preferable for acute and transient symptoms such as acute radiation proctitis (21).

Early results suggest that prostate SBRT with 35–40 Gy in four to five fractions confers rates of biochemical relapse-free survival and late rectal toxicity which are comparable to alternative radiation modalities (22–24). However, little data exist on acute bowel morbidity prior to the 1-month interval, creating a potential for recall bias in patient-reported outcomes. The goal of this study is to accurately delineate the acute rectal morbidity of this treatment modality in the setting of clinically localized prostate cancer.

## Materials and Methods

### Patient Selection and Characteristics

Patients eligible for study inclusion had clinically localized prostate cancer treated on a prospectively conducted IRB-approved institutional QOL protocol (IRB 12-1175). Risk groups were defined using the D’Amico criteria. Other patient and treatment characteristics, such as age, race, Charlson Comorbidity Index (CCI), prostate volume, pretreatment PSA, T stage, Gleason score, hormone treatment, use of anticoagulants and/or antiplatelets, and dose, were acquired from the medical records. Baseline QOL was assessed using the EQ-5D, which contains five general health questions (EQ-5D index) and a visual analog scale (EQ VAS), with higher values indicating better QOL (25).

### SBRT Treatment Planning and Delivery

The CyberKnife robotic radiosurgical system (Accuray Inc., Sunnyvale, CA, USA) was used to deliver SBRT treatments (26). One week prior to simulation, gold fiducial markers were placed within the prostate under ultrasound guidance. For treatment planning, thin cut (1.25 mm) CT images as well as high-resolution magnetic resonance images were acquired and fused. The clinical target volume (CTV) encompassed the prostate and proximal seminal vesicles. The planning target volume (PTV) included a 3 mm (inferior, superior, and posterior) or 5 mm (anterior and lateral) expansion around the CTV. Pelvic lymph nodes were not treated. To account for interfraction and intrafraction prostate motion, fiducial-based tracking was used. Thirty-five or 36.25 Gy was delivered in five fractions (7–7.25 Gy per fraction) to the PTV. The rectum was contoured as a solid structure from the anus (at the level of the ischial tuberosities on axial CT images) to the rectosigmoid flexure. Dose-volume histogram (DVH) analysis was performed using Multiplan (Accuray Inc., Sunnyvale, CA, USA) inverse treatment planning. No more than 1 cc of rectal volume was to receive 36 Gy. Assuming an α/β of 3 Gy for late bowel complications, this is biologically equivalent to approximately 74 Gy administered in 2 Gy fractions. Other rectal DVH constraints included the following: <50% rectal volume was to receive 50% of the prescribed dose, <20% to receive 80% of the dose, <10% to receive 90% of the dose, and <5% to receive 100% of the dose. Typical dose distributions have been previously described (27). To optimize the prostate–rectal wall distance and to reduce intrafraction prostate motion, patients were instructed to adhere to a low-residual diet and undergo enemas prior to simulation and treatment delivery (16).

### Follow-Up and Statistical Analysis

Physician-reported acute bowel toxicity was prospectively assessed at the initial and each subsequent follow-up visit. The National Cancer Institute (NCI) Common Terminology Criteria for Adverse Events version 4.0 (CTCAEv.4) was used to assign toxicity scores. Acute toxicity was defined as having occurred up to 3 months following completion of prostate SBRT. Grade 1 was defined by the CTCAE criteria as rectal discomfort with intervention not indicated. Grade 2 was defined as symptoms (e.g., rectal discomfort, passing blood, or mucus), medical intervention indicated, limiting instrumental activities of daily living (ADL). In this study, patients were generally assigned a grade 2 for requiring a new medication (i.e., steroid suppository or anti-diarrheal). Grade 3 was defined as severe symptoms, fecal urgency, or stool incontinence, limiting self care ADL. In this study, patients were generally assigned grade 3 for requiring a surgical intervention. Cumulative likelihood estimates for acute ≥ grade 2 bowel toxicity were determined using the Kaplan–Meier method.

Acute proctitis was also prospectively reported by patients using the bowel domain of the Expanded Prostate Index Composite (EPIC)-26 questionnaire at baseline (1 hour prior to the first fraction) and at 1 week, 1 month, and 3 months following the final SBRT treatment (28). Patients completed a paper questionnaire at baseline, 1 month, and 3 months. However, to minimize the burden of frequent clinic visits, the 1 week questionnaire was completed *via* a nursing phone interview. The recall period, the time patients are asked to consider when answering questions, was 1 week at the 1 week time point and 1 month for all others. The EPIC-26 bowel domain includes five questions related to individual symptoms (questions 6a–e: urgency, frequency, pain, bloody stool, incontinence) and one question (question 7) related to overall bother (degree of annoyance caused by bowel symptoms).

The EPIC bowel domain and its encompassing questions are scored on a range from 0 to 100, with higher values representing more favorable bowel symptoms. For each EPIC question, the responses were grouped into three clinically relevant categories (no problem, very small to small problem, and moderate to big problem). To statistically compare changes between time points, the levels of responses were assigned a score. The significance of the mean changes in the scores was then assessed by using the Wilcoxon signed-rank test, generating two-sided *p* values. Clinically significant change was assessed by the minimally important difference (MID) in EPIC score, and this was defined as a change of 1/2 standard deviation (SD) from the baseline (29).

## Results

From May 2013 to August 2014, 103 prostate cancer patients were treated on an institutional prospective SBRT QOL protocol. They were ethnically diverse, with 56.3% being of Caucasian ancestry. The median age was 69 years (range, 48–85 years) (Table 1). By D’Amico classification, 19.4% patients were low risk, 65.0% intermediate risk, and 15.5% high risk. A small percentage of patients (16.5%) also underwent androgen deprivation therapy (ADT). Thirty-seven percent also utilized anticoagulants and/or antiplatelets. Approximately half (52%) of the patients were treated with 36.25 Gy in five 7.25 Gy fractions, and 48% were treated with 35 Gy in five 7 Gy fractions.

**Table 1:** **Patient and treatment specifics**.

		Patients *N* = 103 (%)	n
Age (years)	Median 69 (48–85)		
	<60	11.7	12
	60–69	44.7	46
	70–79	35.0	36
	≥80	8.7	9
Race	White	56.3	58
	Black	28.2	29
	Other	15.5	16
Charlson comorbidity index	CCI = 0	61.2	63
	CCI = 1	30.1	31
	CCI ≥ 2	8.7	9
Prostate volume (cc)	Median 36 (13–125)		
Pre-txt PSA (ng/ml)	Median 7 (2.2–50)		
	≤10	75.7	78
	>10 and ≤20	16.5	17
	>20	7.8	8
T stage	T1c	63.1	65
	T2a	20.4	21
	T2b	12.6	13
	T2c	2.9	3
	T3	1.0	1
Gleason score	3 + 3 = 6	32.0	33
	3 + 4 = 7	34.0	35
	4 + 3 = 7	20.4	21
	3 + 5 = 8	1.0	1
	4 + 4 = 8	10.7	11
	4 + 5 = 9	1.9	2
Risk groups (D’Amico)	Low	19.4	20
	Intermediate	65.0	67
	High	15.5	16
Hormone treatment	Yes	16.5	17
	No	83.5	86
Use of anticoagulants/antiplatelets	Yes	36.9	38
	No	63.1	65
Dose (Gy)	35	47.6	49
	36.25	52.4	54

The prevalence of CTCAE-graded GI toxicities at each follow-up time point is illustrated in Table 2, with individual symptoms as well as the highest GI toxicity per patient depicted independently. Bowel frequency and urgency was the most common toxicity, with 23% of patients experiencing grade 2 toxicity at seven days after treatment (Table 2). The cumulative incidence of acute grade 2 GI toxicity was 23%. There were no acute grade 3 or greater toxicities.

**Table 2:** **Prevalence of CTC graded gastrointestinal (GI) toxicities at each follow-up**.

Follow-up time point		Day 7(%)	Month 1(%)	Month 3(%)
Toxicity	Grade
Bowel frequency/urgency	0	51	65	89
	1	26	32	11
	2	23	3	0
Proctitis	0	79	85	98
	1	21	15	2
	2	0	0	0
Rectal bleeding	0	86	91	97
	1	14	9	3
	2	0	0	0
Highest GI	0	43	54	84
	1	34	43	16
	2	23	3	0

**Figure 1:**
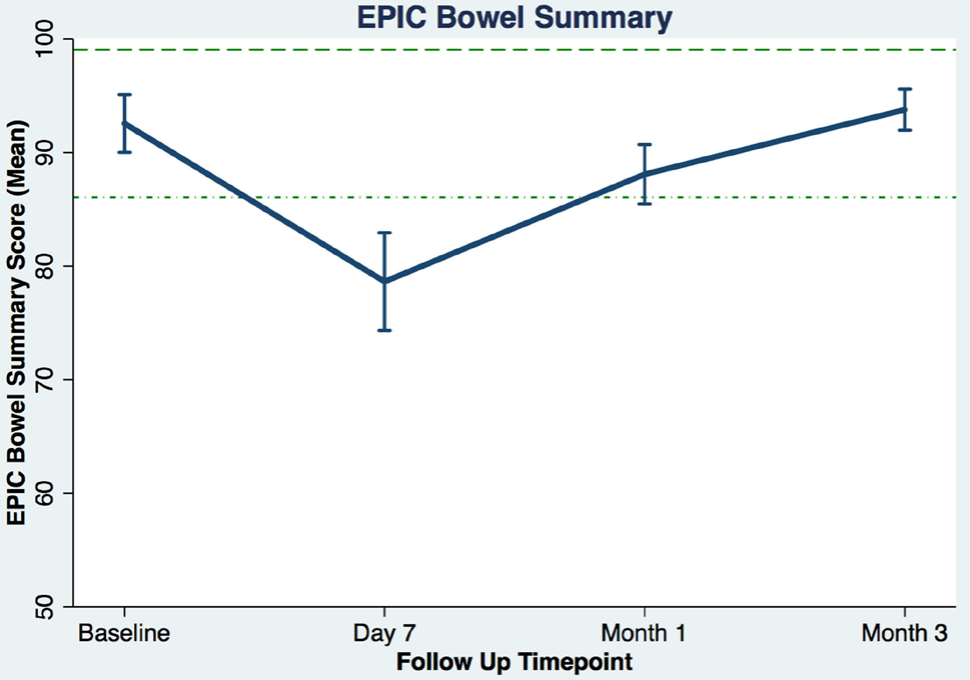
**Average EPIC bowel summary scores (baseline and following SBRT; question 6A-E of EPIC-26)**. Dashed lines represent the minimally important difference (MID) defined as the threshold for clinically significant change in scores (1/2 SD above and below the baseline). Higher EPIC score values (range 0–100) indicate a more satisfactory health-related QOL. Vertical lines at each time point represent 95% confidence interval.

**Figure 2:**
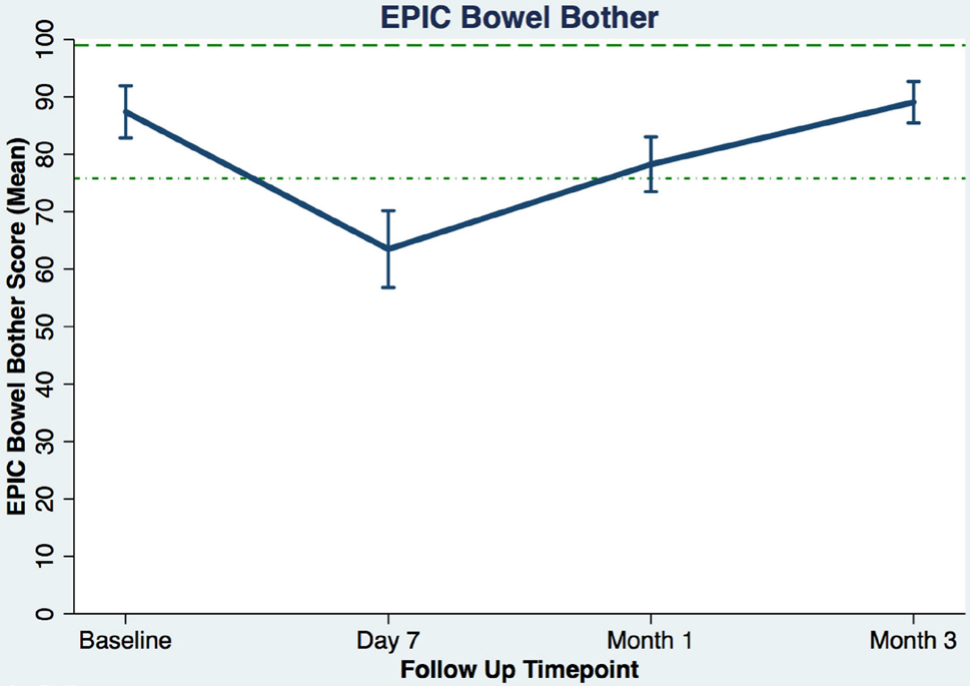
**Overall bowel bother score (baseline and following SBRT; question 7 of EPIC-26)**. Dashed lines represent the minimally important difference (MID) defined as the threshold for clinically significant change in scores (1/2 SD above and below the baseline). Higher EPIC score values (range 0–100) indicate a more satisfactory health-related QOL. Vertical lines at each time point represent 95% confidence interval.

Table 3 shows the baseline EQ-5D, EPIC bowel summary, and EPIC bowel bother scores. Table 4 shows mean changes in EPIC summary scores from baseline to 3 months of follow-up. A transient decline in the EPIC bowel summary score occurred at 1 week (mean change, −13.9; *p* < 0.0001). However, the EPIC bowel summary score returned to baseline 3 months after SBRT (mean change from baseline, +0.03; *p* = 0.9370) (Table 4). While bowel summary score declines at 1 week and 1 month were both statistically significant when compared to baseline (*p* < 0.0001 for both), only the change at 1 week remained below the threshold for clinically significant change (MID = 6.5) (Figure 1). Table 3 shows that the mean baseline EPIC bowel bother score was 87.4. Bowel bother worsened after treatment, with a reduction in mean score to 63.5 at 1 week (mean change, −23.9; *p* < 0.0001) (Table 4). Bowel bother declines at 1 week and 1 month were statistically significant (*p* < 0.0001 and *p* = 0.0002, respectively); however, only the change at 1 week met the threshold for clinically significant change (MID = 11.6) (Figure 2). The EPIC bowel bother score returned to near baseline at 3 months after SBRT (mean change from baseline, −1.8; *p* = 0.6567) (Table 4).

**Table 3:** **Pretreatment quality of life scores**.

	Mean	SD	MID
**Baseline EQ-5D**
EQ-5D index	0.908	0.089	0.045
EQ VAS	80.2	14.87	7.4
**Baseline EPIC-26 domain**			
Bowel summary	92.6	12.99	6.5
Bowel bother	87.4	23.19	11.6

**Table 4:** **Changes in EPIC bowel summary and overall bowel bother scores following SBRT for prostate cancer**.

Domain	1-week post treatment (*n* = 102)			1-month post treatment (*n* = 100)			3-month post treatment (*n* = 82)		
	Mean score change from baseline	SD	p	Mean score change from baseline	SD	p	Mean score change from baseline	SD	p
Bowel summary	−13.9	12.99	*p* < 0.0001*	−4.5	13.18	*p* < 0.0001*	+0.03	8.74	*p* = 0.9370
Bowel bother	−23.9	34.05	*p* < 0.0001*	−9.1	24.01	*p* = 0.0002*	−1.8	17.2	*p* = 0.6567

*Indicates statistical significance.

**Figure 3:**
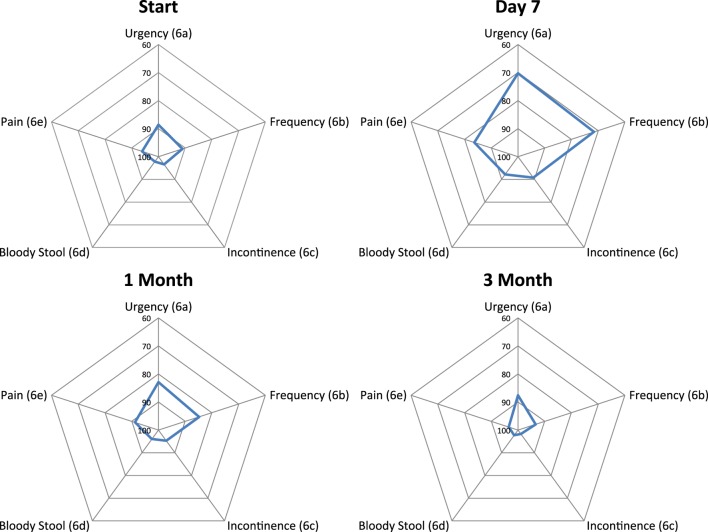
**Radar plots showing the distribution of individual bowel symptoms following SBRT for prostate cancer**. Higher EPIC score values (range 0–100) indicate a more satisfactory health-related QOL. Points farther out from the center indicate higher levels of bother with a given symptom.

Baseline moderate to big bowel symptoms including urgency, frequency, and incontinence were 4.9%, 3.9%, and 1.9%, respectively, with 1% experiencing bloody stool and 1.9% abdominal, pelvic, or rectal pain (Table 5). All bowel symptoms increased at 1 week post-SBRT but returned to near baseline at 3 months (Table 5). At baseline, 31% of patients reported some level of annoyance due to bowel symptoms, with 4.9% of patients feeling that bowel function was a moderate to big problem (Table 6). This is consistent with other studies reporting baseline overall bowel problems in up to 3% of patients undergoing IMRT, proton therapy, or brachytherapy (5, 30). At 1 week following SBRT, 28% of patients felt that their bowel function was a moderate to big problem, and this number declined to 0% by 3 months. Radar plot distribution of individual bowel symptoms at baseline and following SBRT shows the majority of the patients experienced bowel urgency and frequency at 1 week after SBRT, and by 3 months all symptoms subsided to that of the baseline level (Figure 3).

**Table 5:** **Patient-reported bowel symptoms after prostate SBRT as recorded by the EPIC-26 questions 6A (urgency to have a bowel movement), 6B (frequency of bowel movements), 6C (losing control of your stools), 6D (bloody stools), and 6E (abdominal, pelvic, or rectal pain)**.

	Start	Day 7	Month 1	Month 3
**Bowel urgency**				
No problem (%)	71.8	52.0	56.0	65.5
Very small-small problem (%)	23.3	22.5	40.0	29.9
Moderate-big problem (%)	4.9	25.5	4.0	4.6
*p* Value		<0.0001*	0.0056*	0.1128
**Bowel frequency**				
No problem (%)	78.6	55.9	59.0	79.3
Very small-small problem (%)	17.5	19.6	37.0	19.5
Moderate-big problem (%)	3.9	24.5	4.0	1.1
*p* Value		<0.0001*	0.0015*	1.00
**Incontinence**				
No problem (%)	93.2	84.3	86.0	93.1
Very small-small problem (%)	4.9	9.8	12.0	6.9
Moderate-big problem (%)	1.9	5.9	2.0	0.0
*p* Value		0.0066*	0.1531	1.00
**Bloody stools**				
No problem (%)	94.2	86.3	89.0	94.3
Very small-small problem (%)	4.9	8.8	10.0	4.6
Moderate-big problem (%)	1.0	4.9	1.0	1.0
*p* Value		0.0182*	0.2439	0.8203
**Pain (abdominal, pelvic, or rectal)**				
No problem (%)	84.5	72.5	77.0	90.8
Very small-small problem (%)	13.6	13.7	20.0	8.0
Moderate-big problem (%)	1.9	13.7	3.0	1.1
*p* Value		0.0004*	0.0775	0.1294
*N*	103	102	100	87

*Indicates statistical significance.

**Table 6:** **Patient-reported overall bowel bother after prostate SBRT as recorded by the EPIC-26 question 7**.

	Start	Day 7	Month 1	Month 3
**Bowel bother**
No problem (%)	68.9	37.3	43.0	66.7
Very small-small problem (%)	26.2	34.3	51.0	33.3
Moderate-big problem (%)	4.9	28.4	6.0	0.0
*p* Value		<0.0001*	0.0002*	0.6583
*N*	103	102	100	87

*Indicates statistical significance.

## Discussion

What is the appropriate time point and recall period to assess acute bowel symptoms following prostate SBRT such that clinically meaningful symptoms are captured fully and accurately? Early results with prostate SBRT (35–40 Gy in four to five fractions) have suggested very low rates of acute rectal toxicity (22–24). However, such studies did not collect outcomes until 1 month after treatment. As a result of the short SBRT treatment course (1–2 weeks), acute symptoms develop near the end of treatment and last for only a few days, causing potential underestimation of the actual rates of acute morbidity when the assessment is made at one month. On the other hand, the length and burdensome nature of the EPIC-26 would preclude patients from filling out a daily questionnaire. A time point with a 7 day recall period was chosen for this study as providing a balance between the limits of elderly patients’ memory and the burden of filling out time-consuming questionnaires. Thus, to the best of our knowledge, this is the first study to specifically address proctitis 1 week following the completion of prostate SBRT.

Indeed, our results demonstrate that overall acute rectal morbidity is near its maximum several days after the completion of SBRT, improves by 1 month, and returns to baseline by approximately 3 months. Here, we report that 23% of patients experienced ≥ grade 2 acute GI toxicity at 1 week, an incidence which is comparable to those reported for conventional fractionated radiation therapy (9, 10). However, even mild bowel toxicity can adversely affect quality of life (31). Therefore, we utilized the EPIC questionnaire as a sensitive QOL instrument to further assesses bowel quality of life (32–34). The temporal pattern seen in acute bowel QOL and toxicity following SBRT for prostate cancer demonstrated here is comparable to the pattern reported following conventionally fractionated radiotherapy (34–36). The bowel QOL score is at its lowest near the end of treatment, but quickly recovers to the approximate baseline level by 3 months after treatment. Specifically, in our series, most moderate to big problems were seen at 1 week after SBRT, with approximately 25% of patients reporting moderate to big problems with bowel urgency and/or frequency.

Our prior paper reported the prevalence, severity, and overall incidence of bowel frequency and urgency, rectal pain, and rectal bleeding at 1 month following SBRT (24). Because the recall period for the EPIC-26 is 1 month, it was assumed that the 1 month follow-up questionnaire captures bowel morbidity at 1 week. However, based on the results of the current study, it can be concluded that the 1 month questionnaire under-ascertained acute rectal symptoms. Future symptom management trials assessing the impact of interventions on acute bowel morbidity following SBRT should therefore consider including toxicity measurement at 1 week after SBRT.

Currently, there are limited approaches to manage radiation proctitis. Medical treatments such as antidiarrheals or steroid suppositories are commonly administered in response to symptoms with modest benefit (16). Unfortunately, these agents cannot be used as prophylaxis due to significant side effects such as anal irritation and constipation, highlighting the necessity for ongoing clinical research directed at symptom management. In the future, to increase the distance between the prostate and the rectum and thus reduce rectal radiation exposure, we plan to investigate the utilization of absorbable spacers in the context of prostate SBRT (37). Symptom control trials assessing the clinical value of these spacers should evaluate the impact of spacers on acute bowel morbidity 1 week following treatment.

Moreover, a reduction in acute bowel morbidity may improve long-term clinical outcomes. Following conventionally fractionated radiation therapy, non-healing acute proctitis has been shown to directly progress to chronic late bowel morbidity (38). It has been hypothesized that permanent damage occurs when a large area of rectal mucosa is injured and subsequently unable to fully repair (39). If true, reducing the incidence and severity of acute proctitis would lead to reduction of chronic rectal morbidity. The existence of such consequential late effects in the setting of SBRT has yet to be fully characterized. Longer follow-up of this cohort will better elucidate the impact of acute proctitis following SBRT on the incidence of late proctitis.

The present study has several identifiable limitations. In contrast to the paper questionnaires completed prior to each clinic visit, questionnaire completion at the 1 week time point was performed over the phone. Though unlikely, using varied methods of questionnaire completion may have altered the outcomes at 1 week with respect to other time points. It should be noted, however, that previous studies have successfully used third-party phone-survey facilities for collection of quality of life questionnaires in the setting of prostate cancer (5). In addition, the study population was derived from a single institution cohort. This can limit the generalizability of our results to the general population.

## Conclusion

Following prostate SBRT, the incidence and severity of acute radiation proctitis are similar to those seen at the end of a course of conventionally fractionated radiation therapy. Acute radiation proctitis peaks the week following SBRT completion. Therefore, toxicity evaluation and questionnaire completion should be collected at 1 week after treatment. This data should aid in the design of future symptom management clinical trials.

## Author Contributions

Dr. IP is the first author of this manuscript and was responsible for the data acquisition, data analysis, and writing of the manuscript. RC was responsible for the statistical analysis as well as data acquisition. Dr. SC is the principle investigator. All other authors contributed to the data acquisition and analysis.

## Conflict of Interest Statement

SC and BC serve as clinical consultants to Accuray Inc. The Department of Radiation Medicine at Georgetown University Hospital receives a grant from Accuray to support a research coordinator. The other authors declare that they have no competing interests.

## References

[1] Chen RC Zhang Y Chen MH McMahon E Loffredo M McPherson CP Patient-reported quality of life during radiation treatment for localized prostate cancer: results from a prospective phase II trial. BJU Int (2012) 110:1690–5. 10.1111/j.1464-410X.2012.11117.x 22502770

[2] Tucker SL Dong L Michalski JM Bosch WR Winter K Cox JD Do intermediate radiation doses contribute to late rectal toxicity? An analysis of data from radiation therapy oncology group protocol 94-06. Int J Radiat Oncol Biol Phys (2012) 84:390–5. 10.1016/j.ijrobp.2011.11.073 22342302 PMC3952553

[3] Peeters ST Heemsbergen WD van Putten WL Slot A Tabak H Mens JW Acute and late complications after radiotherapy for prostate cancer: results of a multicenter randomized trial comparing 68 Gy to 78 Gy. Int J Radiat Oncol Biol Phys (2005) 61:1019–34. 10.1016/j.ijrobp.2004.07.715 15752881

[4] Vavassori V Fiorino C Rancati T Magli A Fellin G Baccolini M Predictors for rectal and intestinal acute toxicities during prostate cancer high-dose 3D-CRT: results of a prospective multicenter study. Int J Radiat Oncol Biol Phys (2007) 67:1401–10. 10.1016/j.ijrobp.2006.10.040 17241754

[5] Sanda MG Dunn RL Michalski J Sandler HM Northouse L Hembroff L Quality of life and satisfaction with outcome among prostate-cancer survivors. N Engl J Med (2008) 358:1250–61. 10.1056/NEJMoa074311 18354103

[6] Gray PJ Paly JJ Yeap BY Sanda MG Sandler HM Michalski JM Patient-reported outcomes after 3-dimensional conformal, intensity-modulated, or proton beam radiotherapy for localized prostate cancer. Cancer (2013) 119:1729–35. 10.1002/cncr.27956 23436283 PMC3759976

[7] Garg AK Mai WY McGary JE Grant WH Butler EB Teh BS. Radiation proctopathy in the treatment of prostate cancer. Int J Radiat Oncol Biol Phys (2006) 66:1294–305. 10.1016/j.ijrobp.2006.07.1386 17126204

[8] Budaus L Bolla M Bossi A Cozzarini C Crook J Widmark A Functional outcomes and complications following radiation therapy for prostate cancer: a critical analysis of the literature. Eur Urol (2012) 61:112–27. 10.1016/j.eururo.2011.09.027 22001105

[9] Macias V Gonzalez Celador R Marti-Macia C Cigarral C Perez-Romasanta LA. Prognostic factors for acute toxicity in prostate cancer patients treated with high-dose hypofractionated radiotherapy. Clin Transl Oncol (2013) 15:643–51. 10.1007/s12094-012-0987-8 23359176

[10] Gill S Thomas J Fox C Kron T Rolfo A Leahy M Acute toxicity in prostate cancer patients treated with and without image-guided radiotherapy. Radiat Oncol (2011) 6:145. 10.1186/1748-717X-6-145 22035354 PMC3217047

[11] Spratt DE Pei X Yamada J Kollmeier MA Cox B Zelefsky MJ. Long-term survival and toxicity in patients treated with high-dose intensity modulated radiation therapy for localized prostate cancer. Int J Radiat Oncol Biol Phys (2013) 85(3):686–92. 10.1016/j.ijrobp.2012.05.023 22795805 PMC5705018

[12] Arcangeli S Strigari L Soete G De Meerleer G Gomellini S Fonteyne V Clinical and dosimetric predictors of acute toxicity after a 4-week hypofractionated external beam radiotherapy regimen for prostate cancer: results from a multicentric prospective trial. Int J Radiat Oncol Biol Phys (2009) 73:39–45. 10.1016/j.ijrobp.2008.04.005 18538488

[13] Matzinger O Duclos F van den Bergh A Carrie C Villa S Kitsios P Acute toxicity of curative radiotherapy for intermediate- and high-risk localised prostate cancer in the EORTC trial 22991. Eur J Cancer (2009) 45:2825–34. 10.1016/j.ejca.2009.07.009 19682889

[14] Kim DW Cho LC Straka C Christie A Lotan Y Pistenmaa D Predictors of rectal tolerance observed in a dose-escalated phase 1-2 trial of stereotactic body radiation therapy for prostate cancer. Int J Radiat Oncol Biol Phys (2014) 89:509–17. 10.1016/j.ijrobp.2014.03.012 24929162

[15] Pettersson A Johansson B Persson C Berglund A Turesson I. Effects of a dietary intervention on acute gastrointestinal side effects and other aspects of health-related quality of life: a randomized controlled trial in prostate cancer patients undergoing radiotherapy. Radiother Oncol (2012) 103:333–40. 10.1016/j.radonc.2012.04.006 22633817

[16] Yahya S Zarkar A Southgate E Nightingale P Webster G. Which bowel preparation is best? Comparison of a high-fibre diet leaflet, daily microenema and no preparation in prostate cancer patients treated with radical radiotherapy to assess the effect on planned target volume shifts due to rectal distension. Br J Radiol (2013) 86:20130457. 10.1259/bjr.20130457 23995876 PMC3830438

[17] Denham JW O’Brien PC Dunstan RH Johansen J See A Hamilton CS Is there more than one late radiation proctitis syndrome? Radiother Oncol (1999) 51:43–53. 10.1016/S0167-8140(99)00027-4 10386716

[18] O’Brien PC Franklin CI Poulsen MG Joseph DJ Spry NS Denham JW Acute symptoms, not rectally administered sucralfate, predict for late radiation proctitis: longer term follow-up of a phase III trial – trans-Tasman Radiation Oncology Group. Int J Radiat Oncol Biol Phys (2002) 54:442–9. 10.1016/S0360-3016(02)02931-0 12243820

[19] Heemsbergen WD Peeters ST Koper PC Hoogeman MS Lebesque JV. Acute and late gastrointestinal toxicity after radiotherapy in prostate cancer patients: consequential late damage. Int J Radiat Oncol Biol Phys (2006) 66:3–10. 10.1016/j.ijrobp.2006.03.055 16814954

[20] Stull DE Leidy NK Parasuraman B Chassany O. Optimal recall periods for patient-reported outcomes: challenges and potential solutions. Curr Med Res Opin (2009) 25:929–42. 10.1185/03007990902774765 19257798

[21] United States Food and Drug Administration. Guidance for Industry. (2009). Available from: http://www.fda.gov/downloads/Drugs/Guidances/UCM193282.pdf

[22] King CR Collins S Fuller D Wang PC Kupelian P Steinberg M Health-related quality of life after stereotactic body radiation therapy for localized prostate cancer: results from a multi-institutional consortium of prospective trials. Int J Radiat Oncol Biol Phys (2013) 87:939–45. 10.1016/j.ijrobp.2013.08.019 24119836

[23] King CR Freeman D Kaplan I Fuller D Bolzicco G Collins S Stereotactic body radiotherapy for localized prostate cancer: pooled analysis from a multi-institutional consortium of prospective phase II trials. Radiother Oncol (2013) 109:217–21. 10.1016/j.radonc.2013.08.030 24060175

[24] Joh DY Chen LN Porter G Bhagat A Sood S Kim JS Proctitis following stereotactic body radiation therapy for prostate cancer. Radiat Oncol (2014) 9:277. 10.1186/s13014-014-0277-4 25497602 PMC4272823

[25] Torvinen S Färkkilä N Sintonen H Saarto T Roine RP Taari K. Health-related quality of life in prostate cancer. Acta Oncol (2013) 52:1094–101. 10.3109/0284186X.2012.760848 23368678

[26] Lei S Piel N Oermann EK Chen V Ju AW Dahal KN Six-dimensional correction of intra-fractional prostate motion with CyberKnife stereotactic body radiation therapy. Front Oncol (2011) 1:48. 10.3389/fonc.2011.00048 22655248 PMC3356099

[27] Sood S Ju AW Wang H Lei S Uhm S Zhang G Rectal endoscopy findings following stereotactic body radiation therapy for clinically localized prostate cancer. Radiat Oncol (2013) 8:197. 10.1186/1748-717X-8-197 23937800 PMC3751769

[28] Wei JT Dunn RL Litwin MS Sandler HM Sanda MG. Development and validation of the expanded prostate cancer index composite (EPIC) for comprehensive assessment of health-related quality of life in men with prostate cancer. Urology (2000) 56:899–905. 10.1016/S0090-4295(00)00858-X 11113727

[29] Norman GR Sloan JA Wyrwich KW. Interpretation of changes in health-related quality of life: the remarkable universality of half a standard deviation. Med Care (2003) 41:582–92. 10.1097/00005650-200305000-00007 12719681

[30] Hoppe BS Michalski JM Mendenhall NP Morris CG Henderson RH Nichols RC Comparative effectiveness study of patient-reported outcomes after proton therapy or intensity-modulated radiotherapy for prostate cancer. Cancer (2014) 120(7):1076–82. 10.1002/cncr.28536 24382757 PMC4103169

[31] Michalski JM Winter K Purdy JA Wilder RB Perez CA Roach M Preliminary evaluation of low-grade toxicity with conformal radiation therapy for prostate cancer on RTOG 9406 dose levels I and II. Int J Radiat Oncol Biol Phys (2003) 56:192–8. 10.1016/S0360-3016(03)00072-5 12694838

[32] Muanza TM Albert PS Smith S Godette D Crouse NS Cooley-Zgela T Comparing measures of acute bowel toxicity in patients with prostate cancer treated with external beam radiation therapy. Int J Radiat Oncol Biol Phys (2005) 62:1316–21. 10.1016/j.ijrobp.2004.12.083 16029787

[33] Singh AK Menard C Guion P Simone NL Smith S Crouse NS Intrarectal amifostine suspension may protect against acute proctitis during radiation therapy for prostate cancer: a pilot study. Int J Radiat Oncol Biol Phys (2006) 65:1008–13. 10.1016/j.ijrobp.2006.02.030 16730138

[34] Simone NL Menard C Soule BP Albert PS Guion P Smith S Intrarectal amifostine during external beam radiation therapy for prostate cancer produces significant improvements in quality of life measured by EPIC score. Int J Radiat Oncol Biol Phys (2008) 70:90–5. 10.1016/j.ijrobp.2007.05.057 17855015 PMC2267374

[35] Pinkawa M Piroth MD Fischedick K Holy R Klotz J Nussen S Impact of the target volume (prostate alone vs. prostate with seminal vesicles) and fraction dose (1.8 Gy vs. 2.0 Gy) on quality of life changes after external-beam radiotherapy for prostate cancer. Strahlenther Onkol (2009) 185:724–30. 10.1007/s00066-009-2008-6 19899005

[36] Pinkawa M Piroth MD Fischedick K Nussen S Klotz J Holy R Self-assessed bowel toxicity after external beam radiotherapy for prostate cancer – predictive factors on irritative symptoms, incontinence and rectal bleeding. Radiat Oncol (2009) 4:36 10.1186/1748-717X-4-36 19772568 PMC2753361

[37] Mariados N Sylvester J Shah D Karsh L Hudes R Beyer D Hydrogel spacer prospective multicenter randomized controlled pivotal trial: dosimetric and clinical effects of perirectal spacer application in men undergoing prostate image guided intensity modulated radiation therapy. Int J Radiat Oncol Biol Phys (2015) 92:971–7. 10.1016/j.ijrobp.2015.04.030 26054865

[38] Pinkawa M Holy R Piroth MD Fischedick K Schaar S Szekely-Orban D Consequential late effects after radiotherapy for prostate cancer – a prospective longitudinal quality of life study. Radiat Oncol (2010) 5:27. 10.1186/1748-717X-5-27 20377874 PMC2857853

[39] Dorr W Hendry JH. Consequential late effects in normal tissues. Radiother Oncol (2001) 61:223–31. 10.1016/S0167-8140(01)00429-7 11730991

